# Leukemic phase as the initial presentation of ALK‐positive anaplastic large‐cell lymphoma complicated by lactic acidosis

**DOI:** 10.1002/ccr3.6948

**Published:** 2023-02-13

**Authors:** Noriharu Nakagawa, Masahide Yamazaki

**Affiliations:** ^1^ Department of internal medicine Keiju Medical Center Nanao Japan

**Keywords:** ALK‐positive anaplastic large‐cell lymphoma, lactic acidosis, leukemic phase

## Abstract

The leukemic phase of ALK‐positive anaplastic large cell lymphoma (ALCL) is reported to have a poor prognosis. We, herein, report a rare case of the common type of ALK‐positive ALCL complicated by lactic acidosis.

## Graphical abstract

The present case indicates that the leukemic phase of ALCL should be considered in a peripheral blood smear when abnormal lymphocytes are detected, as urgent chemotherapy may rescue life‐threatening LA in the leukemic phase of ALK‐positive ALCL.

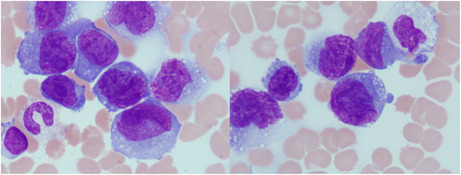

A 47‐year‐old woman visited our hospital with a chief complaint of a low‐grade fever and an inability to move due to fatigue. Her laboratory findings were white blood cell (WBC) count, 21,370/μL; abnormal lymphocytes, 12.0%; lactate dehydrogenase (LDH), 2836 U/L; blood pH, 7.295; HCO_3_, 11.6 mmol/L; and lactic acid, 66 mg/dL. These findings suggested the leukemic phase of malignant lymphoma complicated by lactic acidosis (LA). Computed tomography showed neither lymphadenopathy nor hepatosplenomegaly. She was hospitalized the same day and started on continuous hemodialysis. However, her laboratory findings of the next day of admission were WBC count, 42,540/μL; abnormal lymphocytes, 29.0%; LDH, 5719 U/L; blood pH, 7.317; HCO_3_, 10.8 mmol/L; and lactic acid, 88 mg/dL. She started chemotherapy consisting of etoposide, doxorubicin, cyclophosphamide, vincristine, and prednisone (EPOCH) under continuous hemodiafiltration. LA improved after the first cycle of EPOCH therapy.

May‐Giemsa staining of a peripheral blood smear revealed small to medium‐sized abnormal lymphoid cells (Figure 1). The cytoplasm was basophilic and had vacuoles, along with conspicuous nuclear atypia and distinct nucleoli. The phenotype of the abnormal lymphoid cells was positivity for sCD3(dim+), CD2, CD4, CD7, CD25, and CD30 and negativity for CD5, CD8, and B‐cell phenotype according to a flow cytometric analysis of peripheral blood. Bone marrow aspiration was a dry tap, and bone marrow fluid could not be collected. A bone marrow biopsy showed infiltration by medium to large abnormal cells with immunocytochemistry revealing positive findings for CD30, epithelial membrane antigen (EMA), and anaplastic lymphoma kinase (ALK) (Figure 2) but negative findings for CD34 and terminal deoxynucleotidyl transferase (TdT). The pathological diagnosis of the biopsied specimen proved to be the common type of ALK‐positive anaplastic large cell lymphoma (ALCL).

**FIGURE 1:**
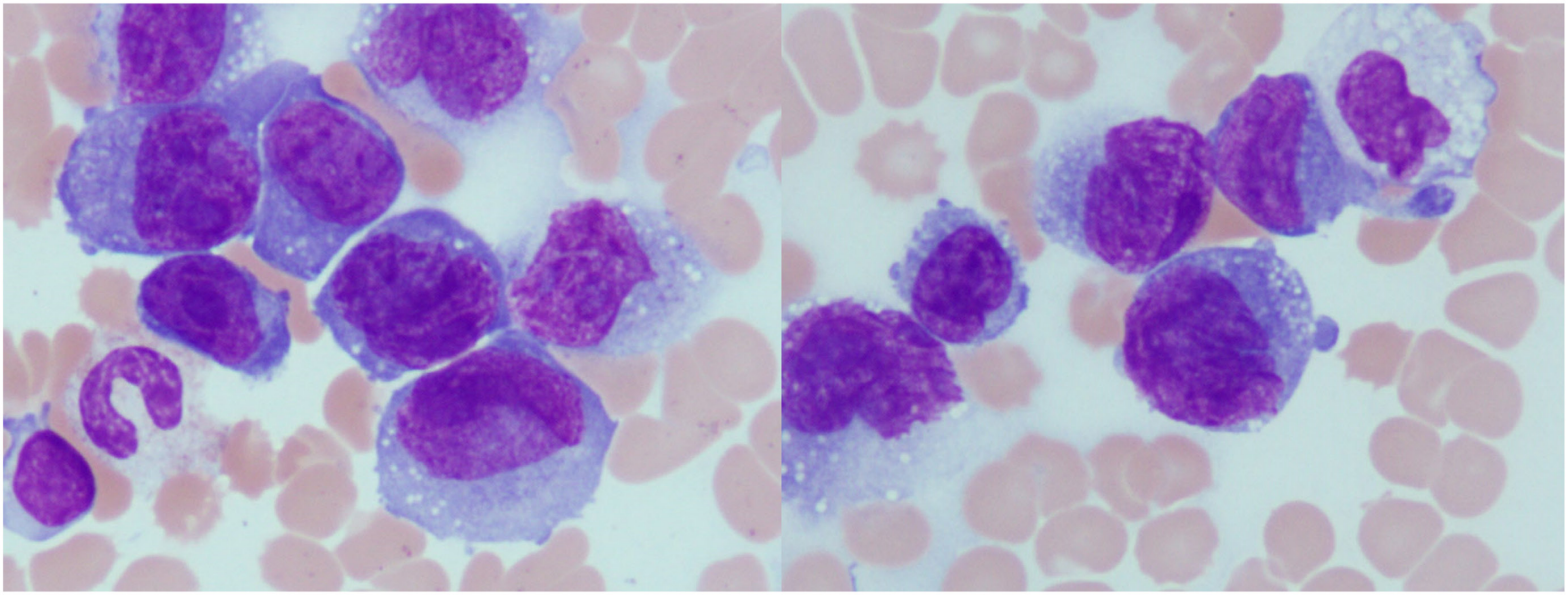
A peripheral blood smear revealed small‐ to medium‐sized abnormal lymphoid cells. The cytoplasm was basophilic and had vacuoles, with conspicuous nuclear atypia and distinct nucleoli (May‐Giemsa, 1000×).

**FIGURE 2:**
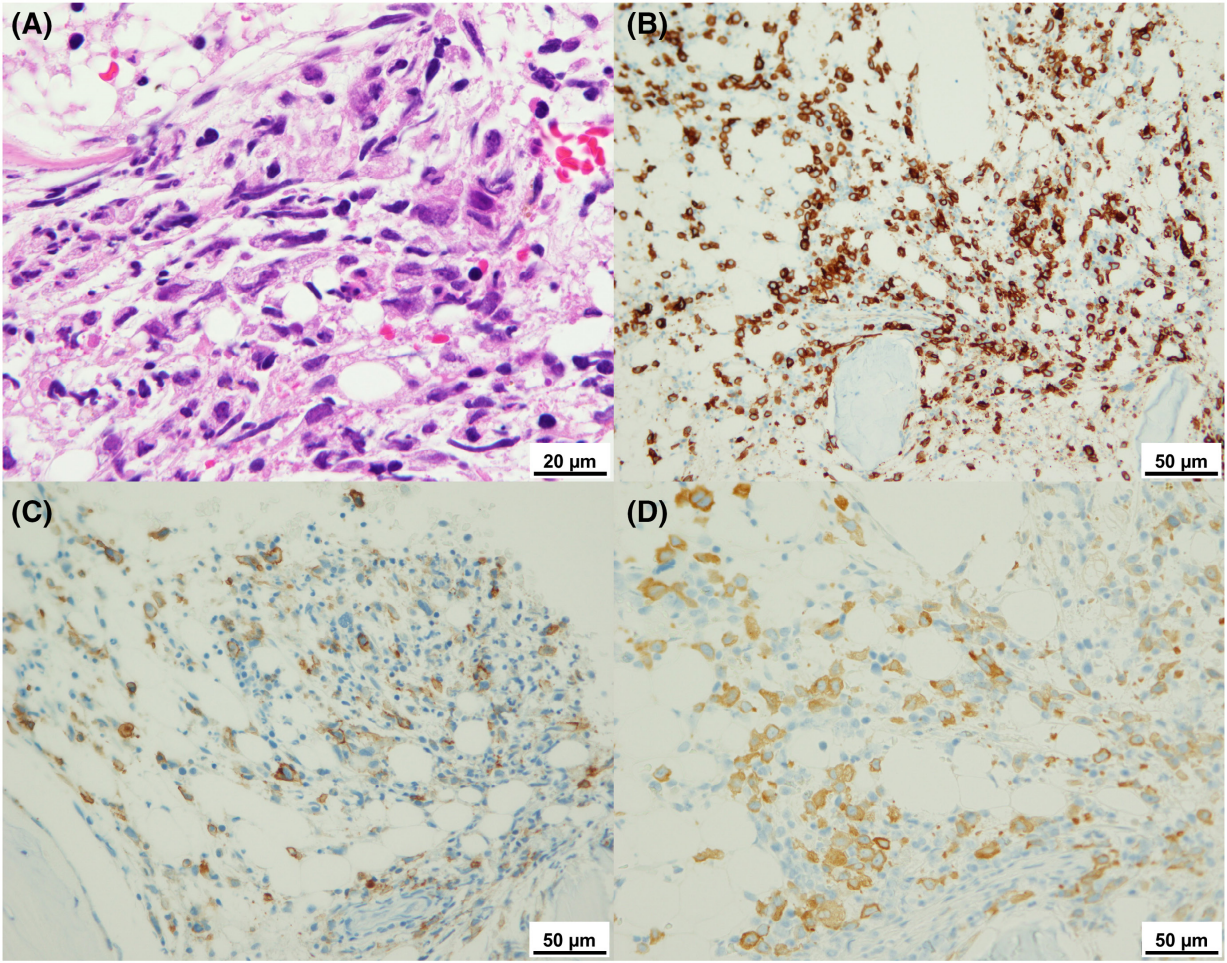
A bone marrow biopsy showed infiltration by medium to large abnormal cells (Hematoxylin and Eosin staining, A) with immunocytochemistry revealing positive findings for CD30 (B), epithelial membrane antigen (C), and anaplastic lymphoma kinase (D).

The patient underwent chemotherapy consisting of brentuximab vedotin, doxorubicin, cyclophosphamide, and prednisone after the first cycle of EPOCH therapy. However, severe headache developed, and the central nervous infiltration of her lymphoma was detected by a lumbar puncture. Papanicolaou staining and May‐Giemsa staining of a cerebrospinal fluid smear revealed abnormal lymphoid cells, and the phenotype was found to be positive for CD4 and CD30. She was started on alectinib and, thereafter, her central nervous symptoms rapidly improved.

Although ALK‐positive ALCL is associated with a favorable prognosis among peripheral T‐cell lymphoma, the leukemic phase of ALCL is reported to have a poor prognosis, especially due to early death.^1^, ^2^ The leukemic phase of ALCL most often is associated with a small‐cell variant type of ALCL.^1^ LA, a fetal complication associated with rapidly growing tumors, occasionally occurs in patients with malignant lymphoma and represents a poor prognostic sign.^3^ We, herein, report a rare case of the common type of ALK‐positive ALCL, not a small‐cell variant, complicated by LA. The present case indicates that the leukemic phase of ALCL should be considered in a peripheral blood smear when abnormal lymphocytes, such as those in the present case, are detected, as urgent chemotherapy may rescue life‐threatening LA in the leukemic phase of ALK‐positive ALCL.

## AUTHOR CONTRIBUTIONS

**Noriharu Nakagawa:** Writing – original draft. **Masahide Yamazaki:** Writing – review and editing.

## FUNDING INFORMATION

The authors received no specific funding for this work.

## CONFLICT OF INTEREST STATEMENT

The authors declare no conflicts of interest in association with the present study.

## CONSENT FOR PUBLICATION

Written informed consent was obtained from the patient to publish this report in accordance with the journal's patient consent policy.

## Data Availability

No data sets were generated or analyzed during this case report.
